# Rattlesnakes and Their Bites

**Published:** 1876-08

**Authors:** 


### *RATTLESNAKES AND THEIR BITES.*

In the course of some notes on the rattlesnake, published in *Forest and Stream*, Dr. J. W. Bailey, of Albany, asserts that this serpent is the most sluggish of the snake family. It never strikes unless in self-defense, excepting just before and after its winter sleep. Of course, the rattlesnake's idea of self-defense is rather broad. Thus, if a person step upon it by the purest accident the snake will make no allowance, but strikes the intruder on the spot. To strike, however, it must be in close coil, with its head erect. It is capable of springing only a little more than half its length, unless it be lying on an inclined plane ; then, by supporting itself entirely on its tail, it can spring much farther. Hogs attack the rattlesnake with impunity, the effect of the poison being probably neutralized by a thick layer of adipose tissue. Dr. Bailey is able to contradict, from his own experience, the statement that serpents do not move about at night ; he has often when riding by moonlight seen them gliding through the grass. The author says that, when the venom of a serpent has entered the circulation, all remedies are unavailing, He has seen a freshly-killed chicken split open and applied to the wound, with good results. In such cases the flesh of the chicken turns green and putrid where it comes in contact with the virus. The most certain remedy, however, is whiskey or brandy used in large quantities—say a quart—immediately. Intoxication is not exhibited until the poison has been counteracted. Sweet-oil, taken in doses of several ounces, is also effectual. Sportsmen camping in Texas are accustomed after pitching their tent, to stretch around it a hair lariat. The short hairs irritate the snake's belly as he attempts to cross the lariat, and he retreats.

ACTING THROUGH THE PORES upon the sources of inflammation GLENN'S SULPHUR SOAP promptly relieves the burning, itching, and other annoyances caused by Salt Rheum, Scald Head, Impetigo, Erysipelas, and other skin diseases, and ultimately removes every vestige of them. Depot, CRITTENTON'S, No. 7 Sixth Avenue.

HILL'S INSTANTANEOUS HAIR DYE is safe as well as speedy.—*Com.*

